# Theoretically proposed optimal frequency for ultrasound induced cartilage restoration

**DOI:** 10.1186/s12976-017-0067-4

**Published:** 2017-11-14

**Authors:** April D. Miller, Anuradha Subramanian, Hendrik J. Viljoen

**Affiliations:** 1 Department of Chemical and Biomolecular Engineering, University of Nebraska-Lincoln, 207 Othmer Hall, Lincoln, NE 68588 USA; 2 Department of Chemistry and Life Science, United States Military Academy, West Point, NY 10996 USA

**Keywords:** Resonant frequency, Mechanical energy density, Cellular deformation

## Abstract

**Background:**

Matching the frequency of the driving force to that of the system’s natural frequency of vibration results in greater amplitude response. Thus we hypothesize that applying ultrasound at the chondrocyte’s resonant frequency will result in greater deformation than applying similar ultrasound power at a frequency outside of the resonant bandwidth. Based on this resonant hypothesis, our group previously confirmed theoretically and experimentally that ultrasound stimulation of suspended chondrocytes at resonance (5 MHz) maximized gene expression of load inducible genes. However, this study was based on suspended chondrocytes. The resonant frequency of a chondrocyte does not only depend on the cell mass and intracellular stiffness, but also on the mechanical properties of the surrounding medium. An in vivo chondrocyte’s environment differs whether it be a blood clot (following microfracture), a hydrogel or the pericellular and extracellular matrices of the natural cartilage. All have distinct structures and compositions leading to different resonant frequencies. In this study, we present two theoretical models, the first model to understand the effects of the resonant frequency on the cellular deformation and the second to identify the optimal frequency range for clinical applications of ultrasound to enhance cartilage restoration.

**Results:**

We showed that applying low-intensity ultrasound at the resonant frequency induced deformation equivalent to that experimentally calculated in previous studies at higher intensities and a 1 MHz frequency. Additionally, the resonant frequency of an in vivo chondrocyte in healthy conditions, osteoarthritic conditions, embedded in a blood clot and embedded in fibrin ranges from 3.5 − 4.8 *MHz*.

**Conclusion:**

The main finding of this study is the theoretically proposed optimal frequency for clinical applications of therapeutic ultrasound induced cartilage restoration is 3.5 − 4.8 *MHz* (the resonant frequencies of in vivo chondrocytes). Application of ultrasound in this frequency range will maximize desired bioeffects.

## Background

Osteoarthritis and cartilage injuries are major biomedical burdens in the United States that affect millions of Americans as cartilage is an avascular, aneural tissue with limited capacity of self-repair. Currently, there are a variety of surgical procedures available to treat articular cartilage, two common methods are microfracture and autologous chondrocyte transplantation (ACI). Microfracture is the first-line treatment for smaller cartilage lesions and involves perforating the subchondral plate to recruit mesenchymal cells (MSC) from the bone marrow [1, 2]. Although the procedure has demonstrated excellent short-term clinical outcomes, the long-term durability of the tissue has shown functional decline as a result of inefficient chondrogenesis of egressed MSCs [3]. ACI and newer cell-based techniques (which include the use of MSCs) are preferred for larger lesions and involves extracting cartilage/cells from the patient followed by re-implantation [2–4]. However, the high cost of the procedure makes it less appealing.

Mechanical and structural cues delivered to the chondrocyte play a central role in the tissue physiology [5]. Thus there is considerable research in techniques to affect the functional adaptation of cartilage [6]. One method known to modulate chondrocytes’ metabolic activity is mechanical stimulus although the mechanisms are only partly understood. The mechanical signals transmitted to the chondrocyte induce extracellular matrix synthesis and maintenance which alters the cartilage structure and composition [7]. A mechanical stimulus believed to trigger signal transduction and induce bioeffects is cellular deformation [8]. Two techniques that have been shown to induce cellular deformation are dynamic compression and ultrasound and both have also been shown to stimulate proteoglycan and collagen II [9–14]. Thus, a beneficial sequential step in all cartilage restoration techniques should involve method(s) to stimulate the chondrocyte to increase the physical function of the restored tissue such as ultrasound.

Low-intensity ultrasound (LIUS) can be delivered using a handheld portable system that can be easily applied in the comfort of patients’ homes. Ultrasound transmits mechanical energy by perturbing cells around their equilibrium position [15] and has been shown to induce cellular deformation in red blood cells, macrophages, MC3T3-E1 and Human Airway Smooth Muscle cells at various frequencies with pressure amplitudes ranging from 12 kPa to 1000 kPa [14, 16–19]. During controlled compression studies, [8] showed that cellular deformation is transmitted to the nucleus through the cytoskeleton, specifically, actin microfilaments. While, [20] confirmed that ultrasound was transmitted to the nucleus by studying the effects of ultrasound on chromatin remodeling. They showed that ultrasound induces chromatin remodeling in chondrocytes and fibroblasts.

Most published in vivo and in vitro cartilage restoration applications use the empirically derived low-intensity pulsed ultrasound (LIPUS) regimens for bone, 1.0–1.5 MHz, and thus leads to variable results [21–25]. To optimize the regime for cartilage repair [26] theoretically determined that suspended chondrocytes have a primary resonance of 5.2 ± 0.8 MHz. Resonance occurs when there is a match between the ultrasound frequency and the elastic properties of the material which generates an increase in the amplitude of displacement [27]. Therefore at this frequency ultrasound will increase the oscillating displacement amplitude in the cell and maximize the mechanical energy coupled to the cell [26]. These findings were further extended to experimental validation using a monolayer of cells and measuring load-inducible gene expression (c-fos, c-jun and c-myc) which showed that ultrasound applied at the resonant frequency of 5 MHz, compared to 2 and 8 MHz, resulted in increased gene expression [26]. Additional experiments, also using a monolayer of cells, confirmed these findings and showed enhanced cellularity and increased matrix and protein synthesis at this resonant frequency [28, 29]. Thus ultrasound maximizes bioeffects when applied at resonance. However, the theoretical model developed to calculate this resonant frequency lacks the biomechanical environment of the in vivo chondrocyte.

The mechanical environment of the chondrocyte plays an important role in cartilage homeostasis. An in vivo chondrocyte following a microfracture procedure is embedded in a blood clot while that following an ACI procedure could be embedded in a hydrogel [30] or native cartilage. An in vivo chondrocyte in native cartilage is embedded in the extracellular matrix (ECM) and surrounded by a narrow region termed the pericellular matrix (PCM) that has a distinct structure and composition that differs from the chondrocyte and ECM [31]. Although the role of the PCM is not fully understood, theoretical models have shown that it plays a major biomechanical role in cell-matrix interactions and serves as a mechanical transducer [32–35]. The contribution of a blood clot, hydrogel or the ECM and PCM may cause a shift in the resonant frequency of an in vivo chondrocyte from that of a suspended chondrocyte. Additionally, osteoarthritis results in degeneration of cartilage and alters the internal structure and material properties [36]. As the resonant frequency is highly dependent on the mass and stiffness, osteoarthritis can also cause a shift in the optimal frequency.

In this study we first present a theoretical model of a suspended chondrocyte to show the effects of continuous ultrasound applied at resonance on cellular deformation. Second, theoretical models of a chondrocyte embedded in a blood clot, embedded in a fibrin hydrogel and surrounded by the PCM and embedded in the ECM are presented to calculate the resonant frequencies of in vivo chondrocytes in different mechanical environments. A range of parameters for the PCM and ECM are reported throughout literature, thus we identify the resonant frequency over a range of mechanical properties and for those properties identified in an osteoarthritis environment.

## Methods: Mathematical modeling

### Ultrasound induced cellular deformation

To theoretically study the effects of frequency on ultrasound induced deformation, the response of a suspended chondrocyte was modeled using the finite element method and facilitated by COMSOL Multiphysics’ built-in Acoustics-Poroelastic Waves Interface (COMSOL Inc., Burlington, MA, USA). Biot’s theory is used to model the cytoplasm and nucleus which is the basis of the governing equations in the Poroelastic Waves Module [37, 38]. Time-harmonic dependence, *p*(*x*, *t*) = *p*(*x*)*e* ^*iωt*^ is assumed which is the case for the application of continuous ultrasound stimulation. The governing equations are given by eqns. 1–2.

1 $$ -\left({\rho}_{av}-\frac{\rho_f^2}{\rho_c\left(\omega \right)}\mathbf{u}\right){\omega}^2\mathbf{u}-\nabla \bullet \left(\boldsymbol{c}:\boldsymbol{\varepsilon} -{\alpha}_B{p}_f\boldsymbol{I}\right)=\frac{\rho_f}{\rho_c\left(\omega \right)}\nabla {p}_f $$

2 $$ \nabla \bullet \left(-\frac{1}{\rho_c}\left(\nabla \boldsymbol{p}-{\boldsymbol{\omega}}^2{\boldsymbol{\rho}}_{\boldsymbol{f}}\boldsymbol{u}\right)\right)-\frac{k_{eq}^2p}{\rho_c}={\omega}^2{\alpha}_B\nabla \bullet \mathbf{u} $$

Eqns. 3–5 define *ρ*_*av*_, the average density, *ρ*_*c*_, the complex density and *k*_*eq*_, the wavenumber. **u** is the displacement vector, *ω* is angular frequency, ***c*** is the elasticity tensor, ***ε*** is the strain tensor and *p* is pressure.

3 $$ {\rho}_{av}={\rho}_d+{\epsilon}_P{\rho}_f $$

4 $$ {\rho}_c=\frac{\tau_{\infty }{\rho}_f}{\epsilon_P}+\frac{\mu_f}{i\omega {k}_P} $$

5 $$ {k}_{eq}^2=\left({\epsilon}_P{\chi}_f+\frac{\alpha_B-{\epsilon}_P}{K_d}\left(1-{\alpha}_B\right)\right){\omega}^2{\rho}_c $$

Louw [26] calculated the resonant frequency of suspended chondrocytes, thus the variables and values used in [26] and defined in Table 1 are used for this study.

**Table 1:** Material properties used in the Biot theory

Cytoplasm			
Bulk Medium			
Bulk Modulus (Pa)	*K*_*d*_	500	[26]
Poisson’s Ratio	*ν*	0.38	[42]
Bulk Density (kg/m^3^)	*ρ*_*d*_	300	[57]
Permeability (m^2^)	*k*_*p*_	7 x 10^−19^	${\mu}_f/\overline{\omega}$ [53]
Porosity	*є*_*p*_	0.75	[57]
Biot-Willis Coefficient	*α*_*B*_	0.9999	*1-K/K*_*s*_
Tortuosity Factor	*τ*_*∞*_	1.2	[58]
Fluid Phase			
Density (kg/m^3^)	*ρ*_*f*_	992.52	[59]
Vicosity (Pa∙s)	*μ*_*f*_	0.7 x 10^−3^	[60]
Compressibility (1/Pa)	χ_*f*_	4.35 x 10^−10^	*1/K*_*f*_
Nucleus			
Bulk Medium			
Bulk Modulus (Pa)	*K*_*d*_	2 x 10^3^	[39]
Poisson’s Ratio	*ν*	0.38	[26]
Bulk Density (kg/m^3^)	*ρ*_*d*_	400	[26]
Permeability (m^2^)	*k*_*p*_	7 x 10^−19^	${\mu}_f/\overline{\omega}$
Porosity	*є*_*p*_	0.65	[61]
Biot-Willis Coefficient	*α*_*B*_	0.9996	*1-K*_*d*_ */K*_*s*_
Tortuosity Factor	*τ*_*∞*_	2	[58]
Fluid Phase			
*See cytoplasm fluid phase*			

**Fig. 1:**
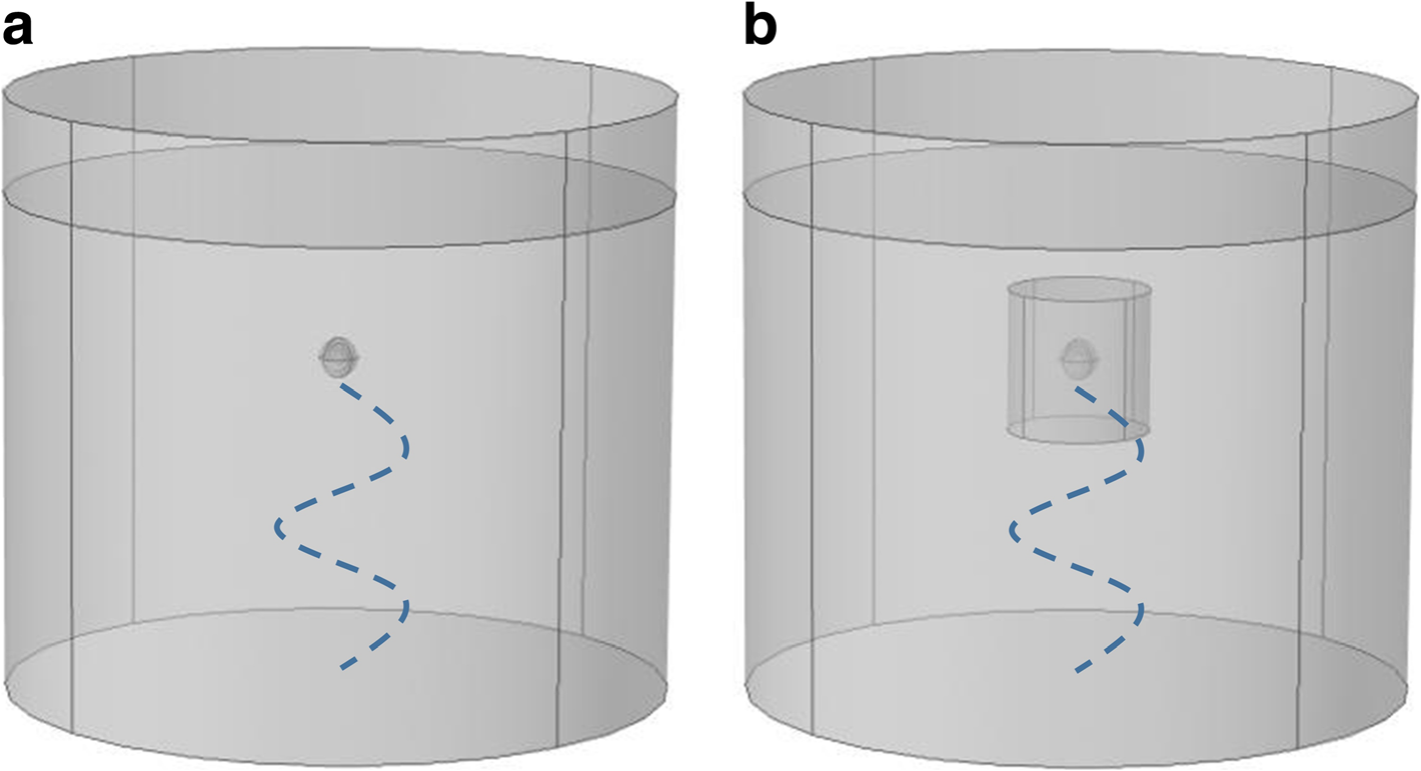
Model Geometry: **a**) A suspended cell (indicated by the small sphere) immersed in growth media. The ultrasound source (14 kPa) is positioned at the bottom and is indicated by the blue dotted line. The cell position is frequency dependent to ensure the position remains at an antinode. **b** A chondron (indicated by the sphere) embedded in the extracellular matrix (indicated by the cylinder) and immersed in growth media. The ultrasound source is positioned at the bottom (blue dotted line). The cell position is frequency dependent

Following [26] the cell was modeled as four concentric spheres; the nucleus, cytoplasm, nuclear envelope and cellular membrane. The cell and nuclear radii were 6.5 and 3.5 μm, respectively [39, 40], and thicknesses of the plasma membrane and nuclear envelope were 15 and 40 nm, respectively [41]. Each domain was modeled as a biphasic medium [39, 42–46]. The geometry consists of a suspended cell in a cylinder (well of a culture plate) filled with growth media. The ultrasound source was positioned below the cell, as shown in Fig. 1a, and the cell’s position is dependent on frequency to ensure the location of the cell was at an antinode. This forces the pressure amplitude at the chondrocyte’s position to remain constant between frequencies to allow for direct comparison. Water properties are used for the growth media and two pressure amplitudes were studied, the amplitude used in [26], 14 kPa, and the amplitude used in [14], 170 kPa. Two boundary conditions were used in this study, a sound hard boundary layer which assumes zero for the normal component of acceleration and cylindrical wave radiation where the outgoing wave leaves with minimal reflection [38].

To minimize the degrees of freedom and computational cost, the geometry was reduced to a height of 3*λ*/4 and a width of *λ*/2, where *λ* is the wavelength. (At 5 MHz in water *λ* = 300*μm* which is approximately 30 cell diameters.) The geometry was meshed using a tetrahedral element which resulted in 40,000–50,000 elements (varies per frequency) and solved on an Intel Core i5 desktop computer with 16 GB RAM. A formation of a standing wave occurred as a result of the geometry dimensions and the water/air interface which is also seen in in vitro experimental setups as a result of the air/polystyrene interface when sonicated from above the cell and the water/air interface when sonicated from below the cell.

### Modeling resonant frequency

The deformation induced by the ultrasound results in the transmission of elastic energy (stored mechanical energy) into the cell. [26] showed that an increase in this stored energy resulted in an increase in load inducible gene expression. Therefore the goal should be to maximize the energy coupled to the cell which occurs if the ultrasound is applied at the chondrocyte’s resonant frequency. The final aim of the study was to calculate the resonant frequency of an in vivo chondrocyte in a blood clot, fibrin hydrogel and under a range of properties for the PCM and ECM. The frequency at which the stored mechanical energy is maximized is the resonant frequency. The stored mechanical energy, *U*, of a cell in an ultrasound field is defined by eq. 6 and has been calculated for both the nucleus and cytoplasm over a range of frequencies (1 MHz – 8 MHz).

6 $$ U=\frac{1}{T}{\int}_0^T\frac{1}{2}\boldsymbol{\sigma} \left(\boldsymbol{t}\right):\boldsymbol{\varepsilon} \left(\boldsymbol{t}\right) dt $$

*T* is the period and ***σ***(***t***) the stress tensor. To further reduce the degrees of freedom and computational cost to conduct the parametric sweep over the range of frequencies the cell was represented as two concentric spheres representing the nucleus and cytoplasm as opposed to four concentric spheres used in the deformation study. Four concentric spheres were used in the deformation study to show the effects of the contribution of the membranes. In the resonance study the aim is to identify the frequency at which the stored mechanical energy is maximized which requires the calculation over a range frequencies and has a higher computational cost. Trials were conducted using four concentric spheres to confirm the resonant frequency did not change. Additionally, an optimization study was conducted to verify the mesh was appropriate and that the resonant frequency did not shift. The mathematical formation for both was the same as that described for the deformation study, detailed above with a pressure amplitude of 14 kPa. The blood clot, fibrin hydrogel, PCM and ECM were assumed to be isotropic biphasic medium. The acoustic properties of blood clots and fibrin are obtained from [47] and shown in Table 2. The mechanical properties of the ECM is known to be depth dependent and the Young’s modulus has been reported to range from approximately 100 kPa to 2 MPa where the middle zone is approximately 500 kPa and deep zone 2 MPa [35, 48, 49]. The material properties of the PCM have been experimentally shown to be approximately constant throughout the tissue depth, however a range of values from 20 to 265 kPa for PCM’s Young’s modulus has been reported [33, 35, 50–52]. The range could be a result of the species, type of sample used, age or measuring technique [31]. Thus this study includes a range of parameters for both the PCM and ECM, listed in Table 2, to study the effects of the mechanical properties on the resonant frequency. The osteoarthritic Young’s modulus was 60% of the normal conditions and the hydraulic permeability is assumed to be homogeneous and isotropic [35].

**Table 2:** Mechanical Properties

Mechanical Properties	Blood Clot	Fibrin	ECM		PCM	
			Normal	Osteoarthritis	Normal	Osteoarthritis
Young’s Modulus (kPa)			500–2000	200–600	1–500	25
Bulk Modulus (Pa)	350.8	475.4				
Shear Modulus (Pa)	228.6	312.5				
Permeability (m^4^/Ns)	1 × 10^−12^	6 × 10^−15^	1 × 10^−15^	2 × 10^−15^	4 × 10^−17^	13 × 10^−17^
Poisson’s Ratio			0.04	0.04	0.04	0.04

The geometry as shown in Fig. 1b, mimics an explant in the well of a culture plate. The ultrasound source was positioned below the tissue and the cell’s position is frequency dependent to allow a direct comparison between frequencies. The ECM was modeled as a cylinder plug with a height of 65 μm and a radius of 32.5 μm which is in agreement with the microscale biphasic model developed by [53] to analyze cell-matrix interactions. Larger heights and widths were also examined to verify the resonant frequency remained the same. Based on [13] measured chondron cross-sectional areas, [25] calculated the typical PCM thickness values to range from 2 to 6 μm, thus the resonant frequency was calculated using the properties listed in Table 2 at 2.5, 6 μm and a 50% increase in thickness for the osteoarthritic conditions [51].

## Results

### Cellular deformation

**Fig. 2:**
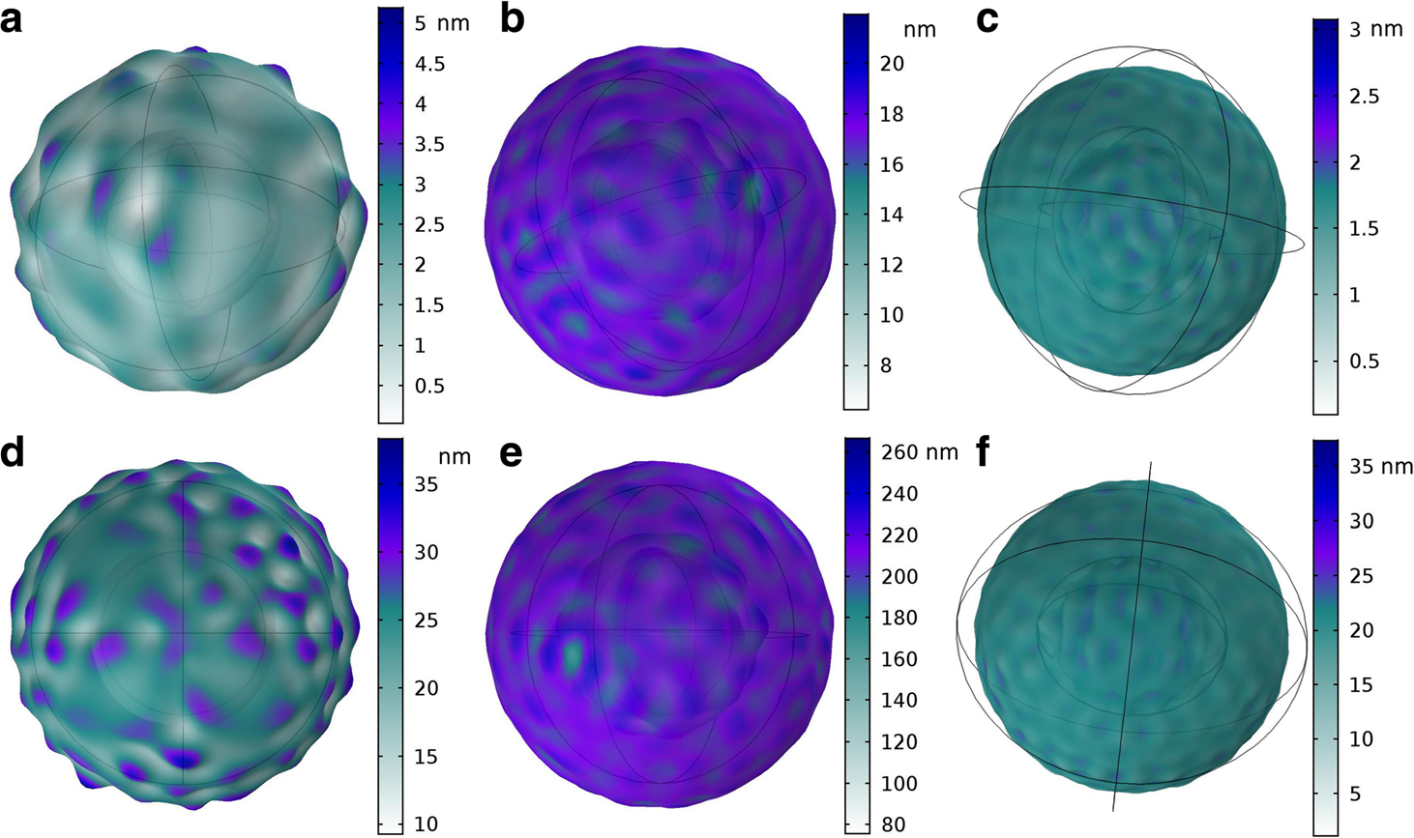
Ultrasound induced cellular deformation, the color represents displacement in nanometers. The frequency and pressure amplitude was varied **a**) 1 MHz; 14 kPa, **b**) 5 MHz; 14 kPa, **c**) 6.5 MHz; 14 kPa, **d**) 1 MHz; 170 kPa, **e**) 5 MHz; 170 kPa, **f**) 6.5 MHz; 170 kPa. (The knobby appearances in A and D are exaggerated to visually see the displacement. The displacement magnitude is depicted by color and is not depicted to scale in the figures)

Ultrasound induced cellular deformation is displayed in Fig. 2. Figure 2a-c are the results of ultrasound application with an initial pressure amplitude of 14 kPa. Figure 2d-f are the results of ultrasound induced cellular deformation with an initial pressure amplitude of 170 kPa.

### In vivo resonant frequency

**Fig. 3:**
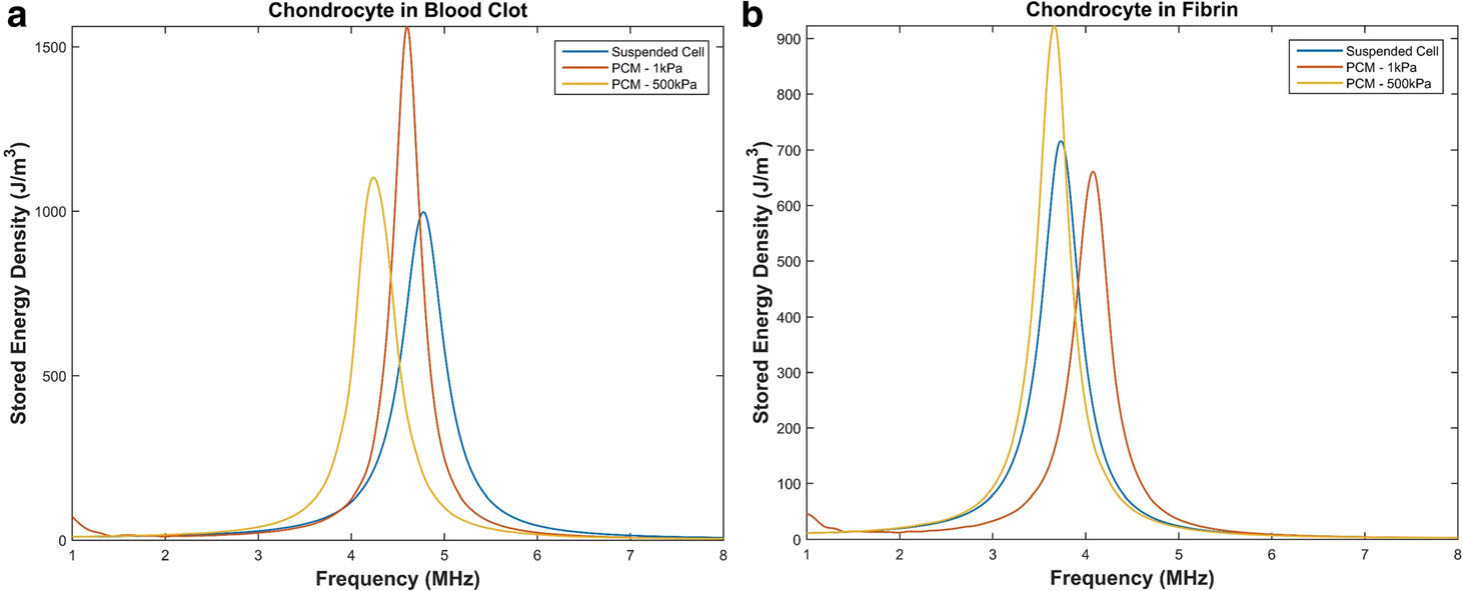
Resonant frequency of a chondrocytes in a blood clot and fibrin. **a** A suspended cell, a chondrocyte surrounded by a PCM with a thickness of 2.5 μm and a Young’s modulus of 1 kPa and a chondrocyte surrounded by a PCM with a thickness of 2.5 μm and a Young’s modulus of 500 kPa embedded in a blood clot. **b** A suspended cell, a chondrocyte surrounded by a PCM with a thickness of 2.5 μm and a Young’s modulus of 1 kPa and a chondrocyte surrounded by a PCM with a thickness of 2.5 μm and a Young’s modulus of 500 kPa embedded in fibrin

**Fig. 4:**
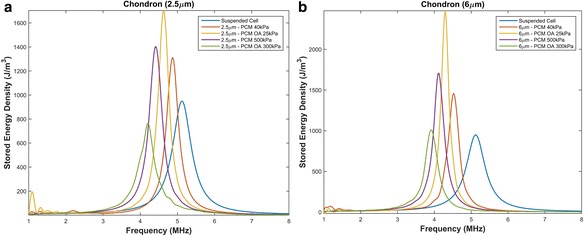
Resonant frequency of chondrons with varying parameters. **a** PCM thickness of 2.5 μm with a Young’s modulus of 40 kPa, 300 kPa and osteoarthritic conditions. **b** PCM thickness of 6 μm with a Young’s modulus of 40 kPa, 300 kPa and osteoarthritic conditions

The resonant frequency of a chondrocyte does not only depend on the cell mass and intracellular stiffness, but also on the mechanical properties of the surrounding medium. An in vivo chondrocyte’s environment differs whether it be a blood clot (following microfracture), a hydrogel or the pericellular and extracellular matrices of the natural cartilage. All have distinct structures and compositions leading to different resonant frequencies. The resonant frequencies of a chondrocyte embedded in a blood clot, which would be the case following microfracture procedures, is shown in Fig. 3a. The effects of the presence of the PCM, with varying properties, surrounded by a blood clot is also shown in Fig. 3a. The resonant frequencies of a chondrocyte embedded in a fibrin hydrogel, which is a type of hydrogel used in ACI procedures [54, 55], is shown in Fig. 3b. The effects of the presence of the PCM, with varying properties, surrounded by a fibrin hydrogel is also shown in Fig. 3b. The effects of the PCM’s mechanical properties for suspended healthy and osteoarthritic chondrons are shown in Fig. 4.

**Fig. 5:**
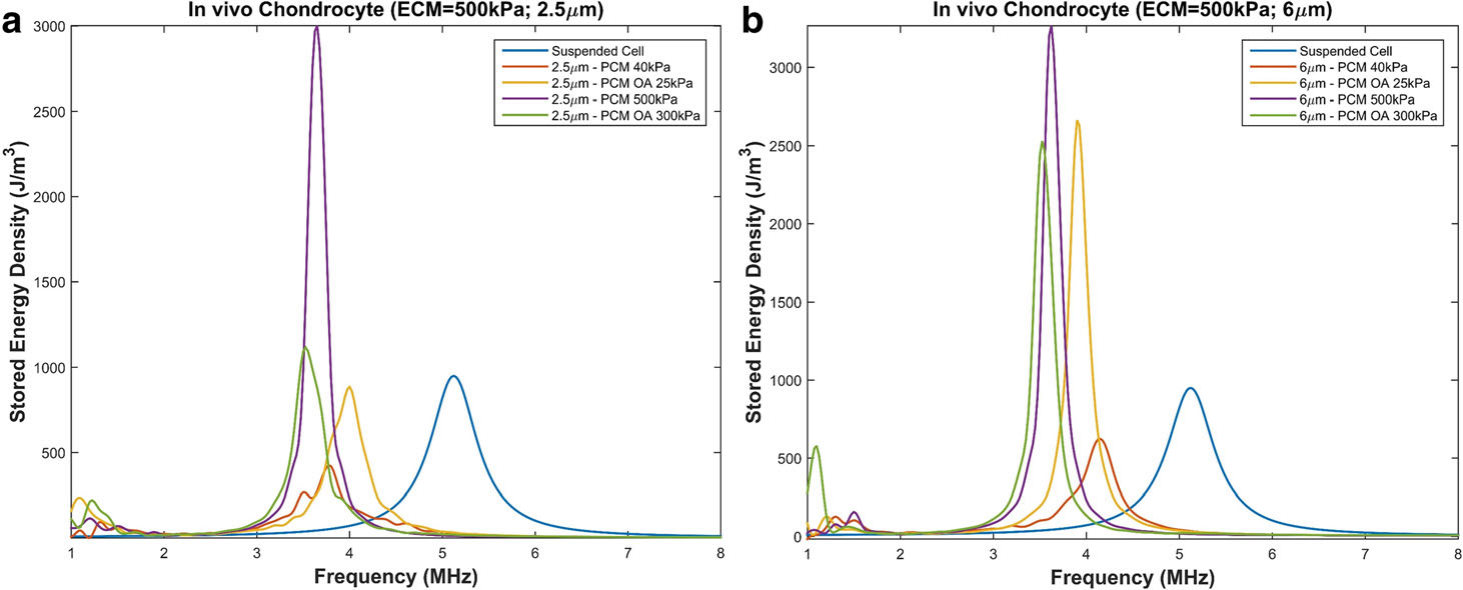
Resonant frequency of chondrons embedded in an ECM with Young’s modulus of 500 kPa with PCM varying parameters. **a** PCM thickness of 2.5 μm with a Young’s modulus of 40 kPa, 300 kPa and osteoarthritic conditions. **b** PCM thickness of 6 μm with a Young’s modulus of 40 kPa, 300 kPa and osteoarthritic conditions

**Fig. 6:**
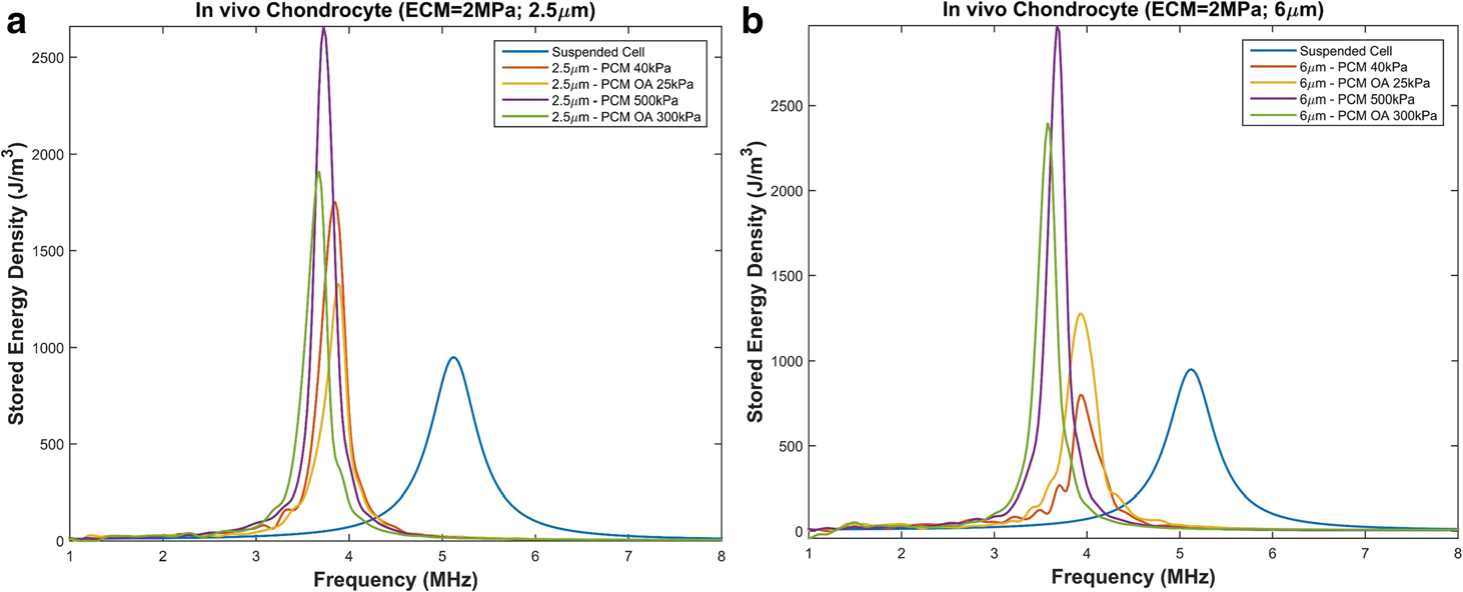
Resonant frequency of chondrons embedded in an ECM with Young’s modulus of 2 MPa with PCM varying parameters. **a** PCM thickness of 2.5 μm with a Young’s modulus of 40 kPa, 300 kPa and osteoarthritic conditions. **b** PCM thickness of 6 μm with a Young’s modulus of 40 kPa, 300 kPa and osteoarthritic conditions

The mechanical properties of the ECM is known to be depth dependent and the Young’s modulus has been reported to range from approximately 100 kPa to 2 MPa where the middle zone is approximately 500 kPa and deep zone 2 MPa [35, 48, 49]. Thus the effects of the ECM’s mechanical properties on chondrocytes’ resonant frequencies are shown in Figs. 5 and 6.

## Discussion

### Cellular deformation

Mizrahi et al. [14] showed that ultrasound applied at 1 MHz with an intensity amplitude of 170 kPa induced an oscillating amplitude of approximately 30 nm in Human Airway Smooth Muscle cells. Figure 2d shows approximately the same deformation in chondrocytes sonicated at the same amplitude of 170 kPa. Comparing the deformation induced by an ultrasound applied at an intensity of 14 kPa, Figs. 2a-c, to the deformation induced by an ultrasound intensity amplitude of 170 kPa, Figs. 2d-f, confirms that increasing the pressure amplitude will increase the deformation. However, if the ultrasound is applied at the resonant frequency the deformation induced by a 14 kPa pressure amplitude, Fig. 2b, is approximately the same as that induced by ultrasound applied at a frequency outside of the resonant bandwidth with an intensity of 170 kPa, Fig. 2d and f. The theoretical model supports the frequency hypothesis theory where ultrasound application at the resonant frequency, even at low-intensities, will cause an even higher deformation than that induced by an ultrasound frequency outside of the resonant bandwidth. This deformation induced by the ultrasound results in the transmission of elastic energy (stored mechanical energy) into the cell. The reader is referred to [56] for additional details on the deformation magnitude of both the nucleus and cytoplasm using a nonlinear model.

### In vivo resonant frequency

#### Blood clot

Microfracture techniques involve perforating the subchondral plate to promote blood flow in order to recruit MSCs from the bone marrow [1, 2]. Differentiation of MSCs to chondrocytes follow, thus chondrocytes would be embedded in a blood clot following a microfracture procedure. The presence of a blood clot results in a slight shift to the left of the resonant frequency from 5.2 MHz to approximately 4.8 MHz, as shown in Fig. 3a. A sequential step would involve the formation of the PCM. The presence of the PCM with properties closer to the cell causes a greater shift, however a PCM with properties closer to native cartilage results in a 1 MHz shift to the left, with a resonant frequency of 4.2 MHz. Ultrasound applied in the range of 4.2–4.8 MHz would be the optimal frequency for ultrasound enhanced cartilage restoration when applied following a microfracture procedure.

#### Fibrin hydrogel

ACI and newer cell-based techniques may involve cells suspended in hydrogels. Therefore, the hydrogel serves as the chondrocyte’s initial mechanical environment. The use of fibrin gels have been shown to serve as a long-term stable hydrogel for cartilage restoration [54, 55], thus we modeled a suspended chondrocyte in a fibrin hydrogel. The presence of a hydrogel shifted the resonant frequency to approximately 3.7 MHz, shown in Fig. 3b. Modeling the cell surrounded by a PCM with a Young’s modulus similar to native cartilage and embedded in the fibrin hydrogel resulted in the same resonant frequency, however, properties closer to the cell resulted in a higher resonant frequency of 4.1 MHz. Therefore, the optimal ultrasound frequency range for ultrasound induced cartilage restoration in conjunction with strategies involving fibrin hydrogels would be 3.7–4.1 MHz.

#### Chondron

A suspended chondron, which includes the chondrocyte and the surrounding PCM, was first modeled separately to see how the presence of the PCM effects the resonant frequency. As shown in Fig. 4 the presence of the healthy PCM causes the chondrocyte’s resonant frequency to shift to the left (a decrease in the resonant frequency). Lower frequencies are beneficial in the application of ultrasound for cartilage restoration as higher frequencies attenuate faster leading to lower intensities at the defect site.

Alexopoulos [51] showed that the Young’s modulus of non-osteoarthritic PCM is approximately 40 kPa. With this modulus, the resonant frequency of a healthy chondron with a thickness of 2.5 μm is approximately 4.9 ± . 1 *MHz* and 4.5 ± . 1 *MHz* for a chondron with a thickness of 6 μm. The larger the PCM thickness the greater the shift in the resonant frequency. Since the thickness of the chondron is not uniform throughout the cartilage structure or in vivo experiments, sonicating suspended chondrons at a frequency within the range of 4.5 − 4.9 *MHz* should maximize the beneficial bioeffects when treating chondrocytes embedded in healthy matrices. However, osteoarthritic chondrons have a greater impact on the resonant frequency with a larger decrease in the resonant frequency. Thus knowledge of the patient’s specific properties or condition is important in designing a specific patient ultrasound regime.

#### Middle zone ECM (Young’s modulus = 500 kPa)

The frequency versus stored energy density of a chondron embedded in the extracellular matrix with a stiffness of 500 kPa is shown in Fig. 5. The presence of the ECM causes an even greater shift towards lower frequencies when compared to a suspended chondron (Fig. 4) and a suspended chondrocyte. The thickness of the PCM has minimal effect on the resonant frequency. It is important to note that linear models, which is used in this study, results in an infinite peak at the resonant frequency. Although there does appear to be an increase in the amount of energy coupled to the cell versus that of a suspended chondrocyte in Figs. 4, 5 and 6 a nonlinear analysis must be conducted to determine the true limits of the resonant bandwidth and peak. Therefore, the peak magnitudes will not be discussed further.

#### Deep zone ECM (Young’s modulus = 2 MPa)

The frequency versus stored energy density of a chondron embedded in an ECM with a stiffness of 2 MPa is shown in Fig. 6. The thickness of the PCM has a greater effect on normal healthy cartilage at lower Young’s modulus. A PCM thickness of 2.5 μm leads to a larger shift to the left than that with a thickness of 6 μm. The thickness does not impact the resonant frequency in the osteoarthritic conditions. From Figs. 5 and 6, one concludes that the stiffness of the ECM has minimal effect on the resonant frequency (comparing mechanical environments of 500 kPa to 2 MPa).

As observed in Figs. 4 and 5 there are peaks in the stored mechanical energy in the range of 1–1.5 MHz that appear to be dampened in stiffer matrices as seen in Fig. 6. The stiffness of the ECM increases with depth of articular cartilage and the modulus of the deep zone is approximately 2 MPa [48, 51]. Thus lower frequencies applied under the typical ultrasound regime for cartilage restoration would affect the middle zone of cartilage and have minimal impact on chondrocytes embedded in the deep zone. This could explain the variable results observed in vivo.

An optimal patient ultrasound regime should be based on the patient specific cartilage mechanical properties which is dependent on the surgical techniques involved. However, the properties are not always available and to physically determine them would be time consuming. Without the knowledge of the patient specific properties, applying ultrasound within the range 3.5 − 4.1 *MHz* for native cartilage should maximize the ultrasound induced bioeffects throughout the entire depth of the cartilage. If a microfracture procedure was conducted than the optimal range would be 4.2–4.8 MHz, however, if a surgical technique involving fibrin hydrogel was used then the optimal range is 3.7–4.1 MHz.

**Fig. 7:**
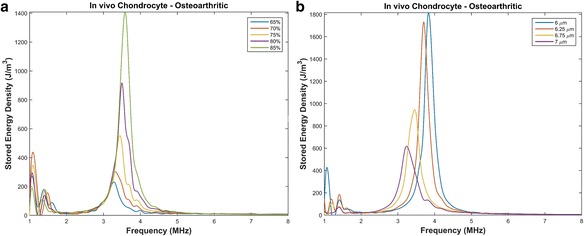
Parameter Analysis. **a** Varying the porosity in an osteoarthritic environment with a PCM thickness of 3.75 μm and a PCM Young’s modulus of 25 kPa and ECM Young’s modulus of 300 kPa. **b** Varying the radius in an osteoarthritic environment with a PCM thickness of 2.5 μm and a PCM Young’s modulus of 40 kPa and ECM Young’s modulus of 500 kPa

The Biot theory involves mechanical properties that are generally not found in literature such as the drained Young’s modulus, thus the solid phase properties were used when not available. As a result a parameter analysis was conducted to understand the sensitivity of the resonant frequency to parameter values. The porosity and radius had the greatest impact on the resonant frequency. The lower the porosity the greater the shift to the left of the resonant frequency and the larger peaks in the lower frequencies. The larger the radius the greater the shift to the left of the resonant frequency. However, the smaller the radius, the greater the peaks are at the lower frequencies as shown in Fig. 7.

## Conclusions

We have theoretically proved the resonant frequency hypothesis for cellular deformation. LIUS applied at the resonant frequency of the cell will induce deformation magnitudes on the order of those induced by high intensity ultrasound applied outside the resonant bandwidth. Additionally, we theoretically determined for the first time the resonant frequency of an in vivo chondrocyte embedded in its mechanical environment. Throughout literature there is a range of properties for the PCM and ECM reported, thus the resonant frequency was identified for a range of parameters and osteoarthritic conditions. Without the knowledge of patient specific mechanical properties an ideal frequency range for ultrasound application to induce maximum bioeffects would be 3.5 − 4.1 *MHz* in native cartilage, 4.2–4.8 MHz when used in conjunction with microfracture techniques or 3.7–4.1 MHz if a fibrin hydrogel is used.
